# No Positive Influence of Ingesting Chia Seed Oil on Human Running Performance

**DOI:** 10.3390/nu7053666

**Published:** 2015-05-15

**Authors:** David C. Nieman, Nicholas D. Gillitt, Mary Pat Meaney, Dustin A. Dew

**Affiliations:** 1 Human Performance Laboratory, Appalachian State University, North Carolina Research Campus, Kannapolis, NC 28081, USA; E-Mails: meaneymp@appstate.edu (M.P.M.); dustindew@gmail.com (D.A.D.); 2 Dole Nutrition Research Laboratory, North Carolina Research Campus, Kannapolis, NC 28081, USA; E-Mail: nicholas.gillitt@dole.com

**Keywords:** alpha-linolenic fatty acid, exercise, cytokines, inflammation

## Abstract

Runners (*n* = 24) reported to the laboratory in an overnight fasted state at 8:00 am on two occasions separated by at least two weeks. After providing a blood sample at 8:00 am, subjects ingested 0.5 liters flavored water alone or 0.5 liters water with 7 kcal kg^−1^ chia seed oil (random order), provided another blood sample at 8:30 am, and then started running to exhaustion (~70% VO_2max_). Additional blood samples were collected immediately post- and 1-h post-exercise. Despite elevations in plasma alpha-linolenic acid (ALA) during the chia seed oil (337%) *versus* water trial (35%) (70.8 ± 8.6, 20.3 ± 1.8 μg mL^−1^, respectively, *p* < 0.001), run time to exhaustion did not differ between trials (1.86 ± 0.10, 1.91 ± 0.13 h, *p* = 0.577, respectively). No trial differences were found for respiratory exchange ratio (RER) (0.92 ± 0.01), oxygen consumption, ventilation, ratings of perceived exertion (RPE), and plasma glucose and blood lactate. Significant post-run increases were measured for total leukocyte counts, plasma cortisol, and plasma cytokines (Interleukin*-*6 (IL-6), Interleukin-8 (IL-8), Interleukin-10 (IL-10), and Tumor necrosis factors-α (TNF-α)), with no trial differences. Chia seed oil supplementation compared to water alone in overnight fasted runners before and during prolonged, intensive running caused an elevation in plasma ALA, but did not enhance run time to exhaustion, alter RER, or counter elevations in cortisol and inflammatory outcome measures.

## 1. Introduction

The essential fatty acid, alpha-linolenic acid (ALA; 18:3*n*-3) can be metabolically converted to long-chain *n*-3 polyunsaturated fatty acids (*n*-3 PUFAs), including eicosapentaenoic acid (EPA; 20:5*n*-3), docosapentaenoic acid (DPA; 22:5*n*-3), and docosahexaenoic acid (DHA; 22:6*n*-3) [1,2]. Enzymatic conversion of ALA to EPA and DHA is relatively inefficient in humans, with less than 1% converted to DHA, 0.3% to 8% to EPA in men, and up to 21% in women [2].

U.S. male and female adults consume about 2.1 and 1.6 g ALA, respectively, well above the Adequate Intake recommendations of 1.6 and 1.1 g day^−1^ [3,4]. A growing number of epidemiologic studies support a variety of health benefits from a higher than normal chronic ALA intake, but studies differ widely regarding influences on disease risk factors including systemic inflammation [5,6,7,8,9,10]. A few long-term ALA supplementation studies indicate that high doses are related to significant decreases in systemic inflammation in at-risk populations [11,12,13]. ALA has not yet been studied as a countermeasure to acute exercise-induced inflammation, but one study showed that older males ingesting 14 g day^−1^ ALA during a 12-week resistance training program experienced a significant decrease in plasma Interleukin*-*6 (IL-6) [14].

After ingestion, ALA is almost completely absorbed and is: (1) oxidized to carbon dioxide and water; (2) incorporated into tissue lipids; or (3) utilized in eicosanoid synthesis [1,2]. After ingestion, [^13^C]-labelled ALA can be detected in plasma within two hours [1]. More than half of ingested ALA is catabolized to carbon dioxide for energy, and the fractional recovery of ingested [^13^C]-ALA as ^13^CO_2_ is almost double that of palmitic, stearic, and oleic acids in human subjects [1]. One possible explanation for the preferential use of ALA for β-oxidation is the greater affinity of carnitine palmitoyl transferase-1, the rate limiting enzyme in mitochondria, for ALA compared to other unsaturated fatty acids [1]. ALA is about 0.7% of total fatty acids in adipose tissue, and we have previously shown using metabolomics that ALA is strongly mobilized during exercise, with plasma levels increasing nearly 6-fold following prolonged, intensive exercise [15]. For all of these reasons, ALA may function as a fuel substrate during long-duration exercise, especially in the later stages when carbohydrate stores are depleted.

Chia seed (*Salvia hispanica* L.) is an oilseed native to southern Mexico and northern Guatemala, and is a rich source of ALA, containing 4.4 g ALA (57% of total fat) per 25 g serving [16,17,18,19,20]. One previous study showed that a mixture of chia seeds and a 6% carbohydrate sports beverage supported 10-km running performance (following a 1-h moderate run) to the same level as an isocaloric volume of the 6% carbohydrate sports beverage alone [21]. Although not measured, these data imply that ALA β-oxidation provided energy to support high intensity running performance. Given the potential for ALA supplementation to support exercise performance and counter post-exercise inflammation, we hypothesized that consuming 7 kcal kg^−1^ chia seed oil 30 min before treadmill running to exhaustion at a set speed (70% VO_2max_) would improve performance and attenuate inflammation compared to water alone in overnight fasted runners.

## 2. Experimental Section

### 2.1. Subjects

Subjects included male (*n* = 16) and female (*n* = 8) runners (ages 24 to 55 years) who competed in long distance road races and were capable of running on treadmills at 70% VO_2max_ to exhaustion. Subjects voluntarily signed an informed consent and agreed to train normally, stay weight stable, and avoid the use of large-dose vitamin/mineral supplements (above 100% of recommended dietary allowances), herbs, and medications (in particular, non-steroidal anti-inflammatory drugs or NSAIDs) during the project. Subjects also agreed to taper their exercise routine prior to each of the two lab exercise sessions as if preparing for a long distance race. Study procedures were approved by the Institutional Review Board at Appalachian State University.

### 2.2. Research Design

This study utilized a randomized (1:1 allocation, random number generator), crossover approach, and subjects engaged in two run-to-exhaustion trials after acute ingestion of flavored water with chia seed oil or flavored water alone (no blinding), with at least a two-week washout period. Subjects completed both arms of the study, and data were analyzed with subjects operating as their own controls using a repeated measures ANOVA within subjects approach.

### 2.3. ALA Bioavailability Study

**Figure 1:**
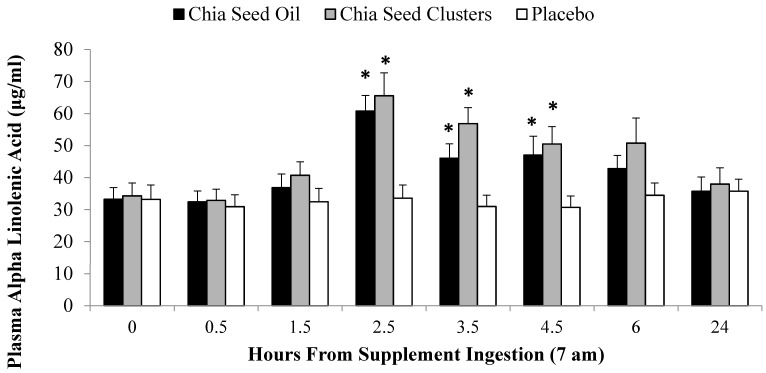
Change in α-linolenic acid (ALA) over time after ingestion of 1.3 mg ALA from milled chia seeds in a snack cluster and chia seed oil (CSO), compared to placebo. * *p* < 0.025, change from pre-supplementation, relative to placebo.

Prior to the current study, little was known regarding the acute plasma ALA response to chia seed or chia seed oil ingestion in overnight fasted human subjects. To provide more information in setting up the research design, a separate cohort of female subjects (*n* = 16, ages 20 to 45 years) reported to the lab after an overnight fast on 3 separate occasions and ingested, in random order by visit, a snack cluster (PL), snack cluster containing 8 g milled chia seed (CS; 1.3 mg ALA), or chia seed oil (CSO; 1.3 mg ALA). The snack clusters were prepared by Dole Foods (Westlake Village, CA, USA), and contained dried cranberries and blueberries, whole rolled oats and oat fiber, brown rice flour, milled chia seeds, dried cane and brown rice syrup, maltodextrin, honey, canola oil, chicory root extract, soy lecithin, and vitamins C and E. A catheter was placed in an antecubital vein, and blood samples were taken at pre-ingestion and post-ingestion (0.5, 1.5, 2.5, 3.5, 4.5, and 6 h) during which the subjects rested and ingested a 550 kcal breakfast at post-1.75 h. Subjects returned to the lab to provide a 24 h sample. Blood samples were analyzed for ALA, EPA, and DHA, and statistical analysis (3 × 8 repeated measures ANOVA) revealed a significant interaction effect for ALA (*p* < 0.001) but not for EPA (*p* = 0.581) or DHA (*p* = 0.445). As shown in Figure 1, ALA in CS (↑82%) and CSO (↑91%) increased significantly within 2.5 h post-ingestion and stayed elevated above PL with some attenuation at 3.5 h, dropping to pre-ingestion levels at 24 h. These bioavailability data indicated that 1.3 mg ALA from ingestion of CS or CSO nearly doubled plasma ALA levels within 2.5 h, stayed elevated for several hs, and then fell to pre-ingestion levels without conversion to EPA or DHA within 24 h. Thus, the exercise trial was designed to ensure that plasma ALA levels from a large chia seed oil dose were elevated near the end of the run-to-exhaustion trial when fatty acids had the potential to be more readily metabolized due to decreasing carbohydrate stores.

### 2.4. Exercise Study Protocol

Two weeks prior to the first running session, subjects completed orientation and baseline testing. Demographic and training histories were acquired with questionnaires. Maximal power, oxygen consumption, ventilation, and heart rate were measured during a graded exercise test (Bruce treadmill protocol) [22] with the Cosmed Quark CPET metabolic cart (Rome, Italy). Body composition was measured with the Bod Pod body composition analyzer (Life Measurement, Concord, CA, USA).

Subjects reported to the lab two times, at least two weeks apart (same day of the week) at 8:00 am in an overnight fasted and rested state (no food or beverage other than water for at least the previous 9 h). Subjects tapered exercise during the previous two days. A blood sample was collected after a 5 min seated rest, and immediately afterwards subjects ingested (in random order across the two lab visits) 0.5 liters flavored water (organic apple sugar free flavor powder, Nature’s Flavors, Orange County, CA, sweetened with Stevia), or the same amount of flavored water with 7 kcal kg^−1^ chia seed oil. The chia seed oil used in this study was 55.3% ALA by weight, and thus 7 kcal kg^−1^ chia seed oil supplied 0.43 g ALA per kg body weight (or 31 g ALA for the average subject in this study). Thirty min later, subjects ran on laboratory treadmills with the speed set at ~70% of VO_2max_. Subjects drank 3 mL kg^−1^ regular water every 15 min during both running trials. Subjects ran as long as possible at a constant speed until exhaustion, and this was defined as the inability of the subject to continue running at 70% VO_2max_ despite verbal support from the laboratory staff). In our laboratory, the mean ± SD difference is 0.068 ± 0.384 h (CV = 11.5%, *R* = 0.771, *p* < 0.001), between repeated treadmill runs to exhaustion at 70% VO_2max_ when separated by 2–4 weeks with subjects (*n* = 47) maintaining normal training. The rating of perceived exertion (RPE) and metabolic measures using the Cosmed CPET system were taken at 15 min, 60 min, 120 min, and at the point of exhaustion. Blood samples were taken immediately pre- and post-exercise, 1-h post-exercise. 

### 2.5. Complete Blood Count, Lactate, Glucose, Cortisol

Complete blood counts (CBC) with a total leukocyte differential count were performed using a Coulter^®^ Ac.TTM 5Diff Hematology Analyzer (Beckman Coulter, Inc., Miami, FL, USA). Shifts in plasma volume due to exercise were calculated using the equation of Dill and Costill [23]. Plasma glucose and lactate were measured pre- and post-exercise using the YSI 2300 STAT Plus Glucose and Lactate analyzer (Yellow Springs, OH, USA). Serum cortisol was measured with an electrochemiluminescence immunoassay (ECLIA) through a commercial lab (LabCorp, Burlington, NC, USA).

### 2.6. Plasma Cytokines

Total plasma concentrations of four inflammatory cytokines [tumor necrosis factor alpha (TNFα), IL-6, Interleukin*-*8 (IL-8), and Interleukin*-*10 (IL-10)] were determined using an electrochemiluminescence based solid-phase sandwich immunoassay (Meso Scale Discovery, Gaithersburg, MD, USA). All samples and provided standards were analyzed in duplicate, and the intra-assay CV ranged from 1.7% to 7.5% and the inter-assay CV ranged from 2.4% to 9.6% for all cytokines measured. Pre-and post-exercise samples for the cytokines were analyzed on the same assay plate to decrease inter-kit assay variability.

### 2.7. Plasma ALA, EPA, DPA, and DHA

Plasma ALA, EPA, and DHA were analyzed as previously described with a few minor modifications [18]. Samples were methylated using 5% methanolic hydrochloric acid and the resulting fatty acid methyl esters were extracted with hexane. Upon solvent evaporation and residue reconstitution, samples were injected into a Thermo Scientific Trace GC Ultra-DSQ II gas chromatograph mass spectrometer for analysis using a DB-23 GC column (60 m × 250 mm × 0.15 mm) from Agilent Technologies (Palo Alto, CA, USA). Identification of the fatty acid methyl esters was based on retention times, standard spectra and NIST library spectra. Quantitation was accomplished by comparison with standard curves established using peak area ratios of the fatty acid methyl ester to that of the internal standard heptadecanoic acid methyl ester.

### 2.8. Statistics

Data are expressed as mean ± SE. Performance data were compared between conditions (water, chia seed oil) using paired *t*-tests. Biomarker data were analyzed using a 2 (condition) × 3 (time) repeated-measures ANOVA, within-subjects design, with changes over time within conditions contrasted between conditions using paired *t*-tests and significance adjusted to *p* < 0.025 after Bonferroni correction.

## 3. Results

Subject characteristics are summarized in Table 1 for the 24 runners (*n* = 16 male, *n* = 8 female) completing all study requirements. Male and female runners did not differ in VO_2max_ (49.5 ± 2.0 and 44.8 ± 2.5 mL·kg^−1^·min^−1^, respectively, *p* = 0.167), or run time and distance to exhaustion in both trials (all *p* > 0.05). Additionally, inflammation and ALA measures before and after the water and chia seed oil trials were comparable for male and female runners (gender × trial × time interaction effects, all *p* > 0.05), and thus genders have been combined for this analysis.

**Table 1:** Subject characteristics (*n* = 24 total, with *n* = 16 males, *n* = 8 females).

Variable	Mean ± SE
Age (year)	38.0 ± 1.7
Height (m)	1.72 ± 0.02
Weight (kg)	71.8 ± 3.0
Body fat %	19.9 ± 1.6
VO_2max_ (mL kg^−1^ min^−1^)	47.9 ± 1.6
Maximal heart rate (beats min^−1^)	180 ± 2.5

Metabolic and performance data measured during the run-to-exhaustion trials under the water and chia seed oil conditions are summarized in Table 2. Performance measures (run time and distance) did not differ between trials, and all metabolic measures were comparable except for a small but significant increase in heart rate during the chia seed oil trial.

**Table 2:** Metabolic and performance data during the run-to-exhaustion trials under water and chia seed oil conditions in trained runners (*n* = 24) (mean ± SE) *.

	Water	Chia Seed Oil	*p*-Value
Time (h)	1.86 ± 0.10	1.91 ± 0.13	0.577
Distance (km)	19.2 ± 1.1	19.8 ± 1.5	0.471
VO_2_ (mL·kg^−1^ min^−1^)	34.4 ± 0.8	34.1 ± 0.7	0.417
HR (beats min^−1^)	154 ± 2.3	156 ± 2.2	0.015
Ventilation (L min^−1^)	69.2 ± 3.4	68.0 ± 3.2	0.317
RPE (average)	13.5 ± 0.3	13.3 ± 0.3	0.438
RER (average)	0.92 ± 0.01	0.92 ± 0.01	0.391
Weight change (kg)	1.4 ± 0.2	1.5 ± 0.2	0.193
Plasma volume shift (%)	−8.6 ± 1.0	−10.7 ± 1.5	0.194
Lactate, pre-exercise	0.53 ± 0.04	0.55 ± 0.04	
Lactate, post-exercise	1.11 ± 0.17	0.98 ± 0.09	0.308
Glucose, pre-exercise	3.98 ± 0.19	4.13 ± 0.12	
Glucose, post-exercise	4.56 ± 0.22	4.47 ± 0.23	0.272

* VO_2_, volume of oxygen consumed; HR, heart rate; RPE, rating of perceived exertion; RER, respiratory exchange ratio (VCO_2_/VO_2_).

Serum cortisol and inflammation measures (total blood leukocytes, plasma IL-6, IL-8, IL-10, TNFα) were elevated post-exercise (all *p* < 0.001) with no trial differences (Table 3).

**Table 3:** Comparison between water and chia seed oil run-to-exhaustion trials for cortisol and inflammation biomarkers in trained runners (*n* = 24) (mean ± SE).

Variable	Pre-Run	Post-Run	1.0-h Post-Run	*p*-Values: Time; Interaction
**Cortisol (nmol L^−1^)**				<0.001; 0.055
Water	364 ± 16.6	433 ± 33.1	359 ± 27.6	
Chia	375 ± 16.6	505 ± 35.9	395 ± 27.6	
**Leukocytes (10^9^ L^−1^)**				<0.001; 0.208
Water	5.33 ± 0.25	16.4 ± 0.9	12.7 ± 0.6	
Chia	5.51 ± 0.28	16.4 ± 0.9	12.7 ± 0.7	
**IL-6 (pg mL^−1^)**				<0.001; 0.368
Water	0.88 ± 0.16	9.37 ± 1.67	5.79 ± 0.92	
Chia	1.02 ± 0.28	8.77 ± 0.69	5.97 ± 0.97	
**IL-8 (pg mL^−1^)**				<0.001; 0.116
Water	5.84 ± 0.49	13.7 ± 1.5	10.7 ± 1.1	
Chia	5.23 ± 0.42	11.8 ± 1.1	7.91 ± 0.74	
**IL-10 (pg mL^−1^)**				<0.001; 0.680
Water	2.21 ± 0.21	38.3 ± 13.7	16.7 ± 4.3	
Chia	2.00 ± 0.19	33.7 ± 10.8	16.9 ± 4.7	
**TNFα (pg mL^−1^)**				<0.001; 0.259
Water	3.86 ± 0.18	4.59 ± 0.26	4.19 ± 0.23	
Chia	3.90 ± 0.16	4.30 ± 0.20	4.10 ± 0.20	

**Figure 2:**
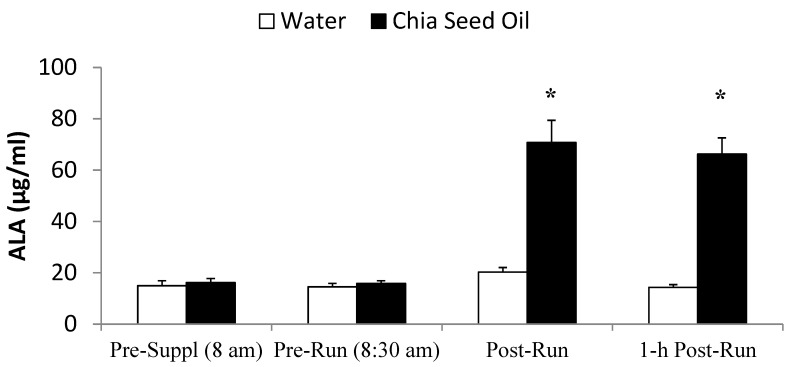
Comparison between water and chia seed oil trials for ALA (interaction effect, *p* < 0.001) in runners (*n* = 24) (mean ± SE). * *p* < 0.025, change from pre-supplementation, relative to placebo.

Plasma ALA was elevated more than 3-fold immediately- and 1h post-run during the chia seed oil trial in comparison to little change following the water trial (interaction effect, *p* < 0.001) (Figure 2). Pre-supplementation and pre-run plasma EPA and DHA, and the overall pattern of change in EPA and DHA did not differ between trials (interaction effects, *p* = 0.618 and 0.831, respectively) (data not shown). 

## 4. Discussion

Contrary to our hypothesis, an acute, large dose chia seed oil supplement providing 0.43 g kg^−1^ ALA to overnight fasted subjects did not improve treadmill run time to exhaustion compared to water alone. Despite a 337% elevation in plasma ALA immediately-post-run during the chia seed oil trial, RER did not differ between trials, indicating no alteration in fatty acid oxidation. The running trials were associated with significant inflammation, with no attenuation when comparing the chia seed oil and water trials.

Chia seed oil is a rich source of ALA, and the participants in this study received 7 kcal chia seed oil per kilogram. This provided a large dose of ALA (~31 g for the average runner), and we reasoned that run time to exhaustion would be increased through enhanced β-oxidation due to greater ALA availability. In the resting state, over half of ALA is metabolized for energy, and there is a preferential use of ALA for β-oxidation compared to palmitic, stearic, and oleic fatty acids [1,2,24,25]. In a metabolomics-based study, we showed that ALA plasma levels increased nearly 6-fold following 75-km cycling, supporting a profound systemic shift in metabolites from the lipid pathway [15]. Nonetheless, greater ALA availability did not lengthen run time or decrease RER as expected in the current study, indicating that ALA supplementation does not improve high-intensity running performance compared to water alone in the overnight fasted state.

Although studies are not consistent, long-term and high ALA intake has been associated with decreased risk of cardiovascular disease [5,6,7,8,26]. Inflammation is a pathophysiologic pathway for cardiovascular disease, and several studies support reduced systemic inflammation in groups with high dietary ALA intake [11,12,13,26]. A cross-sectional study of 353 middle-aged male twins showed that a 1-g increment in habitual dietary ALA intake was associated with 11% lower concentrations of the IL-6 soluble receptor (sIL-6R) [26]. Cytokine production in cultured peripheral blood mononuclear cells (PBMCs) is decreased following high ALA diets in hypercholesterolemic subjects [27]. Increased ALA intake by dyslipidemic subjects decreases C-reactive protein (CRP), serum amyloid A (SAA), IL-6, vascular cell adhesion molecule-1 (VCAM-1), and E-selectin in some [11,12,13] but not all studies [9,16,17,28] utilizing randomized, controlled designs. Little information is available regarding the anti-inflammatory influences of large single doses of ALA, but the current study suggests no attenuation of the acute inflammation induced by intensive exercise. The anti-inflammatory influence of ALA supplementation may in part be due to conversion to EPA, a process that takes at least one week until elevated measures can be detected in blood [18]. This study focused on the acute effects of ALA ingestion on performance and post-exercise inflammation, and further research is warranted to determine if chronic ALA supplementation for two weeks or longer when plasma EPA levels increase would lower post-exercise inflammation.

## 5. Conclusions

Acute ingestion of ALA-rich chia seed oil cannot be recommended as an ergogenic aid during intensive, prolonged running or as a countermeasure to exercise-induced inflammation. Ingestion of 7 kcal kg^−1^ chia seed oil 30 min before running at ~70% VO_2max_ caused a 3.4-fold increase in plasma ALA levels, but provided no discernable benefits for the athletes in this study.
